# A new antimicrobial peptide, Pentatomicin, from the stinkbug *Plautia stali*

**DOI:** 10.1038/s41598-022-20427-w

**Published:** 2022-10-03

**Authors:** Yudai Nishide, Keisuke Nagamine, Daisuke Kageyama, Minoru Moriyama, Ryo Futahashi, Takema Fukatsu

**Affiliations:** 1 Institute of Agrobiological Sciences Ohwashi, National Agriculture and Food Research Organization (NARO), Tsukuba, 305-8634 Japan; 2 Japan Society for the Promotion of Science (JSPS), Tokyo, 102-0083 Japan; 3 National Institute of Advanced Industrial Science and Technology (AIST), Tsukuba, 305-8566 Japan; 4 Department of Biological Sciences, Graduate School of Science, University of Tokyo, Tokyo, 113-0033 Japan; 5 Graduate School of Life and Environmental Sciences, University of Tsukuba, Tsukuba, 305-8572 Japan

**Keywords:** Entomology, Innate immunity, Evolution

## Abstract

Antimicrobial peptides (AMPs) play crucial roles in the innate immunity of diverse organisms, which exhibit remarkable diversity in size, structural property and antimicrobial spectrum. Here, we describe a new AMP, named Pentatomicin, from the stinkbug *Plautia stali* (Hemiptera: Pentatomidae). Orthologous nucleotide sequences of Pentatomicin were present in stinkbugs and beetles but not in other insect groups. Notably, orthologous sequences were also detected from a horseshoe crab, cyanobacteria and proteobacteria, suggesting the possibility of inter-domain horizontal gene transfers of Pentatomicin and allied protein genes. The recombinant protein of Pentatomicin was effective against an array of Gram-positive bacteria but not against Gram-negative bacteria. Upon septic shock, the expression of Pentatomicin drastically increased in a manner similar to other AMPs. On the other hand, unlike other AMPs, mock and saline injections increased the expression of Pentatomicin. RNAi-mediated downregulation of Imd pathway genes (*Imd* and *Relish*) and Toll pathway genes (*MyD88* and *Dorsal*) revealed that the expression of Pentatomicin is under the control of Toll pathway. Being consistent with in vitro effectiveness of the recombinant protein, adult insects injected with dsRNA of Pentatomicin exhibited higher vulnerability to Gram-positive *Staphylococcus aureus* than to Gram-negative *Escherichia coli*. We discovered high levels of Pentatomicin expression in eggs, which is atypical of other AMPs and suggestive of its biological functioning in eggs. Contrary to the expectation, however, RNAi-mediated downregulation of Pentatomicin did not affect normal embryonic development of *P. stali*. Moreover, the downregulation of *Pentatomicin* in eggs did not affect vertical symbiont transmission to the offspring even under heavily contaminated conditions, which refuted our expectation that the antimicrobial activity of *Pentatomicin* may contribute to egg surface-mediated symbiont transmission by suppressing microbial contaminants.

## Introduction

Antimicrobial peptides (AMPs) are ubiquitous in nature, being found in many life forms spanning from microorganisms to humans^1–3^. As of January 2022, 3324 AMPs of various origins have been reported in the AMP database^4^ (https://aps.unmc.edu/AP/). Most AMPs share cationic and amphipathic properties, but differ extremely in length, structural property, and antimicrobial activity spectrum^5,6^.

In insects, AMPs play a pivotal role in humoral immunity by clearing microbial infections, being among the important components of innate immunity in addition to physical and cellular immunities^7,8^. Humoral immunity comprises three stepwise processes, recognition of nonself, signal transduction, and production of effector molecules^9–11^. The upstream pathways of the humoral immune cascade, constituted by microbial recognition and signaling molecules, tend to be highly conserved, which have greatly contributed to our general understanding of the innate immune mechanisms^12^. On the other hand, the downstream effector molecules, including AMPs, are so diverse and taxon-specific that there is much room for exploring novel immune effector molecules and their functions^13^. To fully understand the innate immunity of a certain organism, therefore, it is necessary to investigate the spectrum and effectiveness of each AMP from the effector repertoire of the organism^14,15^. Considering the continuous emergence and spread of pathogenic bacteria resistant to conventional antibiotics^16^, detailed information on novel AMPs may lead to the development of new type of antibiotics^17,18^. Additionally, detailed information on insect immunity may be of use for protecting economically important insects from microbial pathogens.

Horseshoe crabs are relatively large-sized marine arthropods of the family Limulidae^19^. The hemolymph of horseshoe crabs is rich in soluble defense molecules and numerous hemocytes. The coagulation factors constitute a cascade of serine proteases and their inhibitors, sensitively react to bacterial cell wall lipopolysaccharides (LPS) and promptly trigger body fluid clotting, and are commercialized for medical detection of bacterial endotoxins as *Limulus* amebocyte lysate (LAL) reagent^20^. In addition, the hemolymph contains a variety of AMPs, lectins and other presumably defense-related proteins, of which some are well characterized and others are not^21^. *Limulus* endotoxin-binding protein-protease inhibitor (LEBP-PI) represents the latter type, 12 kDa in size, accounting for ~ 1% of hemocyte proteins, purified by LPS affinity column, capable of binding to LPS and bacterial cells, and inhibitory to proteolytic activity of trypsin^22^. However, biological function of LEBP-PI has been elusive: its binding to LPS neither inhibits nor enhances the activation of the clotting system by LPS; its anti-trypsin activity is not affected by the presence of LPS; and it does not exhibit conspicuous antimicrobial activities^22^. Since the first publication in 1991^22^, no study on LEBP-PI has been reported.

Here we report the discovery of an immune-responsive LEBP-PI-related protein in an insect, the brown-winged green stinkbug *Plautia stali* (Hemiptera: Pentatomidae). *P. stali* is known as a notorious agricultural pest infesting various fruits and crop plants^23^, and has attracted much attention in microbiology and evolutionary biology of the gut bacterial symbiosis^24–29^. In this context, *P. stali* has been established as an experimental model system for investigating host–microbe interactions: continuous laboratory maintenance and aseptic rearing are practicaly feasible^28^, efficient gene knockdown by RNAi is applicable^30,31^, and basic information on the innate immune cascade is available^32^. Thus far, Defensins, Hemiptericin and Lysozymes have been identified as immune-responsive AMPs drastically upregulated upon septic shock in *P. stali*^32^. In this study, we identified the LEBP-PI-related gene in the transcriptomic data of *P. stali* as a remarkably upregulated transcript upon septic shock, uncovered its antimicrobial activities against Gram-positive bacteria, designated it as a new AMP “Pentatomicin”, and investigated its structural, evolutionary, immunological and functional aspects in detail.

## Materials and methods

### Insects

Adult insects of *P. stali* were obtained at a forest edge in the National Institute of Advanced Industrial Science and Technology, Tsukuba, Japan, from which an inbred laboratory strain was established. A mass-reared colony of the strain was used as the source of experimental insects. The insects were reared in plastic containers (150 mm in diameter, 60 mm high) with raw peanuts, dry soybeans, and drinking water supplemented with 0.05% ascorbic acid at 25 ± 1 °C under a long-day regime of 16 h light and 8 h dark as described previously^28^.

### Detection and sequencing of Pentatomicin

Total RNAs were extracted from whole adult females approximately 5 days after ecdysis using the RNeasy Mini Kit (Qiagen, Hilden, Germany). Complementary DNAs (cDNAs) were sequenced using Illumina HiSeq 2500 with paired-end 101 bp (Macrogen Japan Corp., Kyoto, Japan), and the generated raw reads were analyzed as previously described^32^.

### Phylogenetic analysis

The deduced amino acid sequences of Pentatomicin of *P. stali* and other organisms were aligned using the Clustal W program implemented in MEGA 5.2^33^. The molecular phylogenetic analyses were conducted by maximum likelihood method using MEGA 5.2 under the WAG + G model that was inferred as the best model. Bootstrap values based on 1000 replications are indicated as percentages on the nodes.

### Construction of the expression vector and recombinant protein expression

To generate the expression vector using In-Fusion HD Cloning Kit (Takara Bio, Shiga, Japan), Pentatomicin-6xHisTag sequence and pGEX-6P-3 (GE Healthcare, Tokyo, Japan) sequence lacking GST except for the first 5 codon were amplified by PCR. The primer sequences are shown in Supplemental Table 1. These PCR products were ligated using In-Fusion HD Cloning Kit. The pGEX-6P-Pentatomicn-Histag plasmid was transformed into *Escherichia coli* BL21 (DE3) competent cells (Merck, Tokyo, Japan) to express Pentatomicn peptide. After incubation at 37 °C for 4 h in LB medium, the recombinant *E. coli* was cultured for 20 h at 18 °C with 0.1 mmol/l isopropyl-β-d-thiogalactopyranoside (IPTG).

### Purification of recombinant protein

The cultured recombinant *E. coli* cells were lysed by ca. 40 mg of chicken lysozyme (Sigma-Aldrich Japan, Tokyo, Japan), and ultrasonic vibration. The lysates were centrifuged, and the supernatants were subjected to TALON metal affinity Resin (Takara Bio) for recombinant protein purification, basically following the manufacturer’s instructions except for the elution buffer, wherein 250 mM imidazole instead of 150 mM imidazole was dissolved. The purified recombinant protein was dialyzed using Spectra/Por membrane (Fisher Scientific, Pennsylvania, USA) to remove inadequate salts. The dialyzed recombinant protein was subjected to SDS-PAGE and quantification using ImageJ (https://imagej.nih.gov/ij/). As quantification standards, 2000, 1000, 500, 250, and 150 µg/µl of bovine serum albumin solutions were used.

### Assay of antimicrobial activity

The antimicrobial activity assay was performed using eight bacterial strains. *E. coli* EPI300 was purchased from ARBROWN (Tokyo, Japan). *Enterobacter cloacae* (MAFF No. 811101)^34^ and *Bacillus subtilis* (MAFF No. 301702)^35^ were obtained from National Agriculture and Food Research Organization (NARO) Genebank, Japan. *Micrococcus luteus* (NBRC No. 13867), *Staphylococcus aureus* (NBRC No. 12732), *Enterococcus faecalis* (NBRC No. 100480) and *Enterococcus faecium* (NBRC No. 100486) were obtained from Nite Biological Resource Center (NBRC), Japan. The cultivable gut symbiont, *Pantoea* sp. C, used in this study was isolated from *P. stali* midgut^25^. The bacterial media are shown in Supplemental Table 2. The bacterial culture was mixed with the recombinant protein and incubated at 25 °C for 24 h. Bacterial growth was measured using Picoexplorer (Yamato Scientific Co., Tokyo, Japan), by which 200 µl of culture medium was measured at the wavelength of 575–660 nm.

### Septic shock experiments

Adult females approximately 5 days after ecdysis were injected using glass capillary tubes (size: 100 µl; Drummond, Alabama, USA). As controls, we performed mock injection, i.e., pricking with a glass capillary without injecting any solution, and saline injection, i.e., injecting 5 µl of 0.9% sodium chloride (physiological saline for insects). The females were injected with ca. 5 µl of 0.9% saline or 10^8^ cells/µl heat-killed bacteria (*E. coli* or *M. luteus*) into the ventral septum between the thoracic and abdominal segments^32^. Total RNA was extracted from the fat body excised 1, 4, 8 or 24 h after injection.

### Quantitative RT-PCR

Total RNA samples were prepared from abdominal fat bodies using TRIzol (Life Technologies Japan Ltd, Tokyo, Japan), reverse-transcribed into complementary DNA using High-Capacity cDNA Reverse Transcription Kit (Life Technologies Japan Ltd, Tokyo, Japan). Quantitative reverse transcribed PCR (qRT-PCR) of *Pentatomicin* was conducted with primers, which were designed based on RNA sequencing data (Supplemental Table 1), using the LightCycler 96 SYBR Green Master (Roche Diagnostics, Tokyo, Japan). In the test of expression levels of Pentatomicin in different body parts of *P. stali*, mainly female bodies were used, but male-specific organs, genitalia and testis, were obtained by dissecting males.

### RNAi experiments on adults

To knockdown the gene expression, adult females approximately 5 days after ecdysis were injected with ca. 3 µl of double-stranded RNA (dsRNA) solution (100 ng/µl) into the ventral septum between the thoracic and abdominal segments. dsRNA was synthesized from a PCR product using primers listed in Supplemental Table 1 and RiboMAX Large Scale RNA production kit (Promega, Madison, USA). The insects were injected with 3 µl of 10^8^ cells/µl heat-killed *E. coli* or 3.3 ng/µl peptidoglycan of *E. coli* (Invivogen, San Diego, USA) 3 days after dsRNA injection, and then subjected to RNA extraction on the following day. The extracted RNA samples were subjected to quantitative RT-PCR (as above) to quantify the expression levels of *Pentatomicin*. pEGFP-L1 (Takara Bio) was used as a template for enhanced green fluorescent protein (EGFP). The concentrations of dsRNA were estimated using Nanodrop Lite (Thermo Fisher Scientific, Wilmington, DE, USA).

### Survival assay on adult insects upon bacterial challenge

Male adults approximately 1 week after ecdysis were subjected to dsRNA injection as described above. Three days after dsRNA injection, the adults were challenged with living *E. coli* or *S. aureus*.

To prepare bacterial cells for injection, overnight bacterial cultures were diluted by 40 times and cultured for 6 h at 37 °C for *E. coli* and at 31 °C for *S. aureus*. Then, the bacterial cells were centrifuged and washed with PBS. Preliminary experiments were conducted, in which the injected doses were selected such that at least some control individuals (EGFP dsRNA injected) were killed. The bacterial cell titers injected into the insects, which were estimated in terms of colony-forming unit, were, on average, 1.3 × 10^10^ (high concentration), 8.6 × 10^9^ (medium concentration), and 4.8 × 10^9^ (low concentration) for *E. coli*, and 5.0 × 10^8^ (high concentration), 3.6 × 10^8^ (medium concentration), and 2.3 × 10^8^ (low concentration) for *S. aureus*, respectively.

The survival rates upon bacterial challenge were observed every 24 h and the figures for survival rates were drawn using EzR^36^. *P*-values were calculated using pairwise comparisons using Log-Rank test.

### RNAi experiments on eggs and nymphs

Sexually mature female adults (2 or 3 weeks after ecdysis) were injected with dsRNA of EGFP or *Pentatomicin*. Then, the eggs laid by injected females were collected daily to analyze the effects of RNAi using qRT-PCR. The effects of RNAi on newly hatched nymphs from those eggs were also tested using qRT-PCR.

### Survival assay on eggs and nymphs

To estimate the biological role of *Pentatomicin* in eggs, the hatching rate of eggs laid by females injected with dsRNA of EGFP or *Pentatomicin* was inspected. Furthermore, to test the effects on vertical transmission of the obligate symbiont, the rate of attaining the 3rd instar was observed during 10 days after hatching, on the ground that most aposymbiotic nymphs died at 2nd instar or remained at 2nd instar with growth retardation^24,25,28^. In this experiment, in addition to placing the eggs in a clean dish with sterilized water and peanuts, four different treatments were applied to the eggs for experimental challenge of facilitating microbial contamination: (i) placing the eggs on 20-fold diluted *S. aureus* culture medium; (ii) soaking the eggs once in *S. aureus* culture medium (not diluted) and then placing them in a clean dish; (iii) placing the eggs on muddy water; and (iv) soaking the eggs once in muddy water and then placing them in a clean dish. The muddy water was made by suspending 100 g of soil from the forest floor with 400 ml of distilled water.

### Diagnostic PCR to detect symbiotic bacteria

DNA samples were extracted from 2nd instar nymphs 3 days after ecdysis using Wizard Genomic DNA Purification Kit (Promega). PCR was conducted using Ex-taq (TaKaRa Bio) and the primers as described previously^25^ (sequences shown in Supplemental Table 1).

### Statistical analysis

Steel–Dwass test was conducted by R3.4.2 (https://www.R-project.org/) and Mann–Whitney *U* test was done by EzR^36^.

## Results and discussion

### Identification of Pentatomicin from *P. stali*

**Figure 1:**
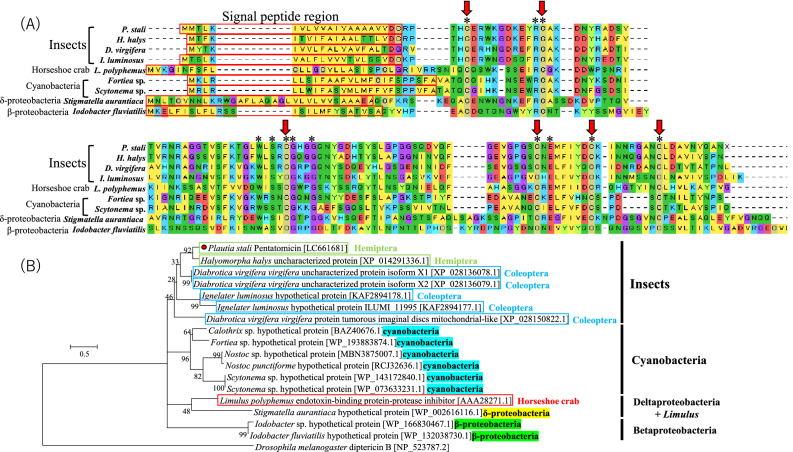
(**A**) Aligned deduced amino acid sequences of Pentatomicin from nine organisms. Red boxes indicate signal peptide regions. Conserved amino acid residues are shown by asterisks, of which conserved cysteine residues are highlighted by red arrows. (**B**) Phylogenic tree of Pentatomicin from *P. stali* and the orthologous genes from other organisms based on the deduced amino acid sequences. The tree was inferred from 71 aligned amino acid sites using the maximum likelihood method (WAG + G model). Bootstrap probabilities based on 1000 replications are indicated on the nodes. A red circle highlights Pentatomicin from *P. stali*. Accession numbers are indicated in brackets. Group labels are shown on the right side of the phylogeny.

RNA sequencing was conducted using a control adult insect without treatment, an adult insect injected with heat-killed *E. coli*, and an adult insect injected with heat-killed *M. luteus*^32^. Compared with the non-treated control, 37 of 114,190 contigs were commonly upregulated upon septic shocks by *E. coli* and *M. luteus* (over 30 times). The upregulated genes included previously reported AMPs such as Defensin1 and Hemiptericin (Supplemental Table 3). Here, we focused on a transcript of a potential immune effector that was highly upregulated upon septic shocks and exhibited notable sequence and structural similarities to LEBP-PI of the *Limulus* horseshoe crab^22^. The 560 bp transcript with polyadenylation signal and poly-A tail (Fig. S1) encoded a polypeptide of 126 amino acid residues with a secretion signal peptide on the C terminus (Fig. 1A). We designated the protein as “Pentatomicin” after the pentatomomorphan stinkbug *P. stali*.

### Structural features and diversity of Pentatomicin and allied proteins

Pentatomicin gene identified from the transcriptome of *P. stali* was subjected to BLASTp searches against non-redundant protein sequences database in NCBI. Among diverse insects, a close Pentatomicin ortholog was identified from a stinkbug *Halyomorpha halys* (Hemiptera: Pentatomidae). In addition, several Pentatomicin orthologs were detected from a leaf beetle *Diabrotica virgifera* (Coleoptera: Chrysomelidae) and a click beetle *Ignelater luminosus* (Coleoptera: Elateridae). Meanwhile, although ample genomic and transcriptomic data are available for many hemipterans, coleopterans and other insect groups, Pentatomicins were not found from them. Notably, LEBP-PI derived from the *Limulus* horseshoe crab^22^ was identified as Pentatomicin-allied. While this protein was reported to bind to LPS and inhibit protease activities, its antimicrobial activities has not been documented^22^. Furthermore, Pentatomicin-allied proteins were detected from diverse prokaryotes encompassing Cyanobacteria, Betaproteobacteria and Deltaproteobacteria (Fig. 1B). These small proteins, 125–159 amino acid residues in size, shared six conserved cysteine residues whereas the other regions exhibited considerable sequence diversity (Fig. 1A). The six cysteine residues were not found in conventional AMPs like defensin, hemiptericin, drosomycin, diptericin and bacteriocin (Fig. S2). More accurate information will be obtained by detailed structural analysis, such as mass spectrometry, which awaits further research.

### Evolutionary dynamics of Pentatomicin and allied proteins

Molecular phylogenetic analysis revealed that Pentatomicin and allied proteins may be classified into the following groups: (i) Insect group, (ii) Cyanobacteria group, (iii) Betaproteobacteria group, and (iv) Deltaproteobacteria + *Limulus* group (Fig. 1B). It should be noted, however, that the groupings are not necessarily robust with low statistical supports (especially Insect group and Deltaproteobacteria + *Limulus* group) presumably due to small size and variability of the sequences. In the insect group, the close relationship of the Pentatomicins between *P. stali* and *H. halys* must reflect the common ancestry of the gene in the Pentatomidae. On the other hand, the intermingled relationship of the Pentatomicin-like sequences from *D. virgifera* and *I. luminosus* suggested dynamic evolutionary trajectories of the genes that may entail both ancestral and recent gene duplications. The clustering of the deltaproteobacterial sequence and the *Limulus* sequence was suggestive of an inter-domain horizontal gene transfer event. Taken together, the sporadic distribution of Pentatomicin and allied proteins across arthropods and diverse bacteria strongly suggest that Pentatomicin has been moving around within and between the prokaryotes and the eukaryotes.

### Antimicrobial activity of Pentatomicin

**Figure 2:**
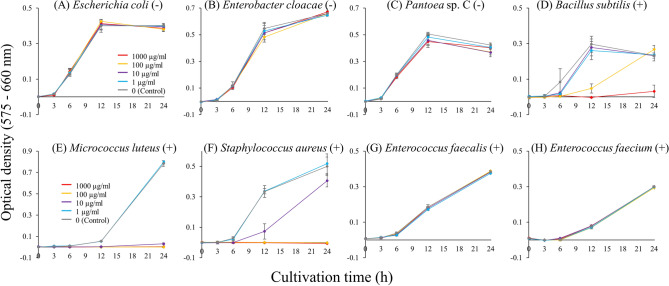
Analysis of the activity of Pentatomicin against eight bacterial species. (**A**) *Escherichia coli*. (**B**) *Enterobacter cloacae*. (**C**) *Pantoea* sp. C, the gut symbiont of *P. stali*. (**D**) *Bacillus subtilis*. (**E**) *Micrococcus luteus*. (**F**) *Staphylococcus aureus*. (**G**) *Enterococcus faecalis*. (**H**) *Enterococcus faecium*. Each bacterial culture was mixed with the recombinant Pentatomicin and incubated at 25 °C for 24 h. Bacterial growth was measured for 200 µl of culture medium at the wavelength of 575–660 nm. Values represent the means ± SD of three replicates.

We expressed Pentatomicin protein in *E. coli* cells and purified the recombinant protein, 13.6 kDa in size, for antimicrobial activity assay (Fig. S3). Of eight bacterial species tested, all three Gram-negative bacteria (*E. coli*, *Enterobacter cloacae* and *Pantoea* sp. C symbiotic to *P. stali*^25^) were not affected, whereas three of five Gram-positive bacteria (*Bacillus subtilis*, *Micrococcus luteus* and *Staphylococcus aureus*, but not *Enterococcus faecalis* and *Enterococcus faecium*) were significantly suppressed by the presence of Pentatomicin (Fig. 2; Fig. S4). The minimum inhibitory concentrations (MIC) were 100–1000 µg/ml for *B. subtilis*, 1–10 µg/ml for *M. luteus*, and 10–100 µg/ml for *S. aureus*. These results demonstrated that Pentatomicin is an AMP of *P. stali* with broad activity against Gram-positive bacteria, except for *Enterococcus* spp.

### Expression patterns and regulatory pathways of Pentatomicin

**Figure 3:**
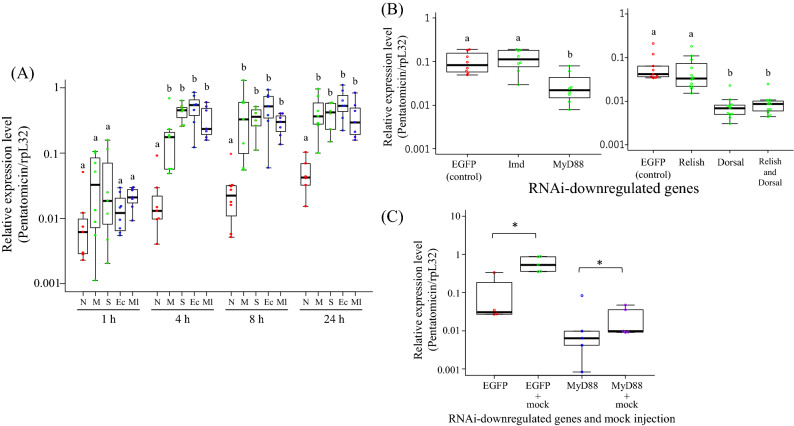
The expression patterns of *Pentatomicin* in adult fat bodies. (**A**) The effects of septic shock treatments on the expression levels of *Pentatomicin*. Adult females were subjected to the following treatments: *N* no treatment, *M* mock injection, *S* saline injection, *Ec* heat-killed *E. coli* injection, *Ml* heat-killed *M. luteus* injection. Quantitative RT-PCR was performed for the samples harvested 1, 4, 8, and 24 h after injection (n = 5–8). Different letters show statistically significant differences in each time point (Steel–Dwass test: *P* < 0.05). (**B**) Effects of RNAi knockdown of immune pathway genes on septic shock-induced upregulation of *Pentatomicin*. Adult insects were injected with heat-killed *E. coli* 3 days after dsRNA injection and subjected to RNA extraction on the following day (n = 9–12). Different letters show statistically significant differences (Steel–Dwass test: *P* < 0.05). (**C**) Effects of RNAi knockdown of immune pathway genes on mock injection-induced upregulation of *Pentatomicin*. Adult insects were injected with a clean needle 3 days after dsRNA injection and subjected to RNA extraction on the following day (n = 4–5). Statistical analysis was done by Mann–Whitney *U* test (*P* < 0.05).

Among diverse insects in general, microbial challenges promptly induce the expression of an array of AMPs in specific tissues like fat bodies^37,38^. Here, the expression levels of Pentatomicin upon and after septic shock were monitored using fat bodies of female adults of *P. stali* (Fig. 3A). The expression levels of Pentatomicin increased nearly 100-fold 4 h after injection and on with heat-killed *E. coli* or *M. luteus*. Though less promptly than the bacterial injections, mock and saline injections induced the expression of Pentatomicin at comparable levels (Fig. 3A). Note that the other AMPs of *P. stali* are not so responsive to mock and saline injections^32^. These results suggest that *Pentatomicin* is highly sensitive to low doses of contaminated bacteria and/or mechanical injury.

The expression of AMPs is regulated by signaling pathways^39^. In insects, Toll pathway and immune deficiency (IMD) pathway mainly regulate microbe-induced production of AMPs^11,40,41^. We conducted RNAi-mediated suppression of the immune regulatory genes and examined how the expression levels of Pentatomicin upon septic shock are affected. The upregulation of Pentatomicin was inhibited by the RNAi-mediated suppression of Toll pathway genes *MyD88* and *Dorsal* (Mann–Whitney *U* test; *P* < 0.05; ca. 70% and 90% lower than control, respectively), whereas no inhibition was observed with the RNAi-mediated suppression of IMD pathway genes *Imd* and *Relish* (Fig. 3B). In *P. stali*, at least 5 AMPs are known to be regulated by both IMD and Toll pathways, which are more or less entangled^32^. By contrast, *Pentatomicin* seems to be regulated by the Toll pathway preferentially (Fig. S5).

Meanwhile, we point out the possibility that Toll pathway may be not the main and direct regulator of *Pentatomicin*. The reduction in *Pentatomicin* expression by Toll pathway blockage was not so drastic in contrast to the other AMPs: for instance, RNAi knockdown of Toll pathway component, *MyD88*, results in ca. 95% downregulation of *Defensin2*^32^. The upregulation of *Pentatomicin* was induced even by mock or saline injection only, which is extremely sensitive in comparison with the other AMPs. This property of Pentatomicin might be relevant to the fact that it is not a typical insect AMP but is allied to LEBP-PI known as a LPS binding protein of the *Limulus* horseshoe crab^22^.

Finally, we investigated whether the effects of mock injection on Pentatomicin could be suppressed by downregulating the Toll pathway. The results showed that RNAi of MyD88 did not cancel the effect of mock, and the expression of Pentatomicin was upregulated by mock injection (Mann–Whitney *U* test; *P* < 0.05; Fig. 3C). This strongly suggests that some other effect, not via the Toll pathway, is responsible for the upregulation of Pentatomicin by mock injection.

### Biological role of Pentatomicin for adult survival

**Figure 4:**
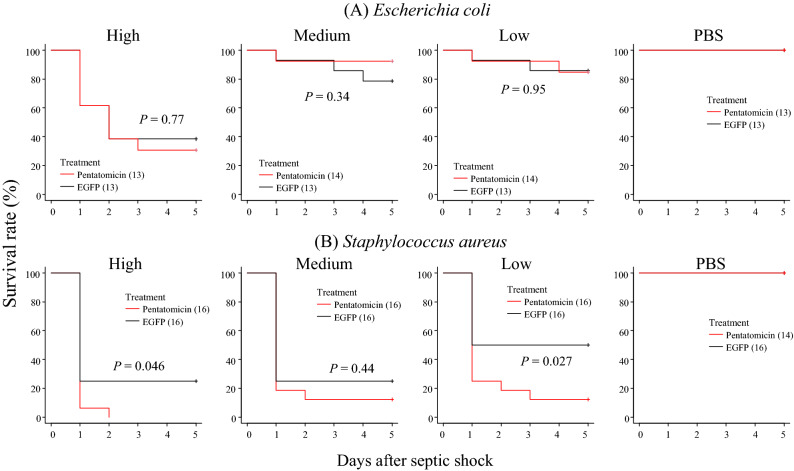
The effects of *Pentatomicin* suppression on adult survival against septic challenge by *E. coli* (**A**) or *S. aureus* (**B**). The average numbers of bacterial cells injected into *P. stali* adults were 1.3 × 10^10^ (high concentration), 8.6 × 10^9^ (medium concentration) and 4.8 × 10^9^ (low concentration) *E. coli* cells per insect, and 5.0 × 10^8^ (high concentration), 3.6 × 10^8^ (medium concentration) and 2.3 × 10^8^ (low concentration) *S. aureus* cells per insect. Red lines indicate the survival curves of *Pentatomicin* dsRNA-injected insects, whereas black lines show the survival curves of EGFP dsRNA-injected insects. The number of individuals is given in parentheses. *P* values are calculated by pairwise comparisons using Log-Rank test.

To examine whether Pentatomicin plays an immunological role in *P. stali*, we injected dsRNA of *Pentatomicin* into adult males approximately 1 week after ecdysis. Quantitative RT-PCR confirmed that the dsRNA injection efficiently suppressed the upregulation of *Pentatomicin* by peptidoglycan injection (Fig. S6). When these insects were challenged by bacterial infections, Gram-positive *S. aureus* caused significantly higher mortality than control (Fig. 4B), although medium concentration of *S. aureus* was not affected. On the other hand, Gram-negative *E. coli* induced no significant mortality in comparison with control (Fig. 4A). These results are consistent with the antimicrobial spectrum of the recombinant *Pentatomicin* (see Fig. 2) and suggest that *Pentatomicin* contributes to host insect survival via immune defense against Gram-positive bacterial pathogens.

### Discovery of high Pentatomicin expression in developing eggs

**Figure 5:**
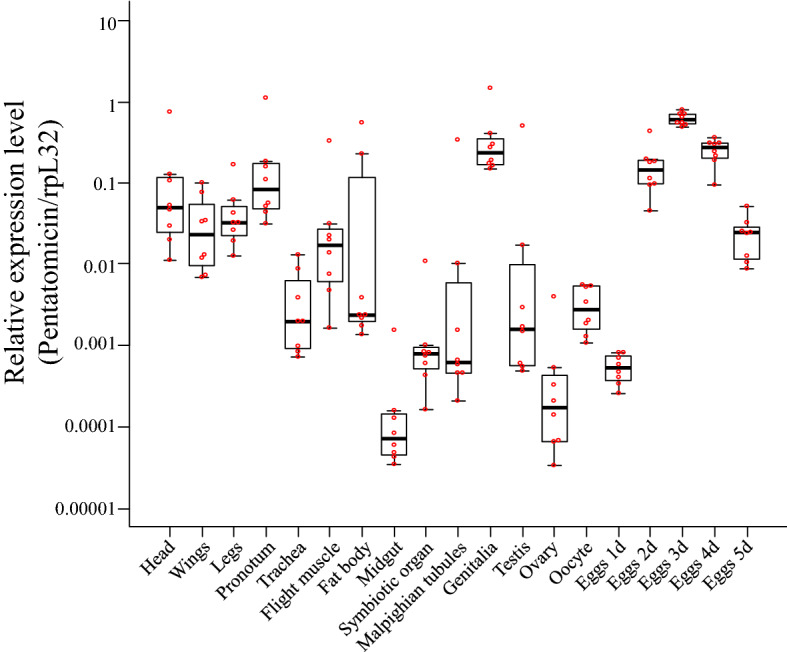
The expression levels of *Pentatomicin* in different body parts of *P. stali*. Eight individuals were examined for each body part. The highest mean expression was observed in eggs 3 days after oviposition. The top and bottom bars around the boxes indicate the maximum and minimum values of the sample (except for outliers which are shown by circles). The top and bottom lines of the boxes indicate the 75% quartile and 25% quartile of the samples, respectively. The thick lines inside the boxes indicate medians.

In an attempt to explore the roles of *Pentatomicin* in more depth, we examined the expression levels of *Pentatomicin* in different body parts of *P. stali*. Relatively high expression levels in head, wings, legs, pronotum, and genitalia (Fig. 5) seemed likely due to the presence of fat bodies and/or epithelia in these body parts where AMPs are synthesized. Unexpectedly, the highest expression levels of *Pentatomicin* were detected in eggs 3 days after oviposition, and the relatively high expression levels persisted in eggs 4 and 5 days after oviposition (Fig. 5). This finding was surprising because egg contents are encased in a hard shell and thus considered as aseptic. In *Drosophila*, it has been established that 16 AMPs (Defensin, Drosocin, DiptericinA, DiptericinB, AttacinA, AttacinB, AttacinC, AttacinD, Metchnikowin, Drosomycin, Drosomycin-like1, CecropinA1, CecropinA2, CecropinB, CecropinC, and Andropin) consistently exhibit no or very low expression in eggs, although AMP-like genes called Bomanins are expressed in late embryonic stages (https://flybase.org/)^42–44^.

### Biological role of Pentatomicin for embryonic development?

**Figure 6:**
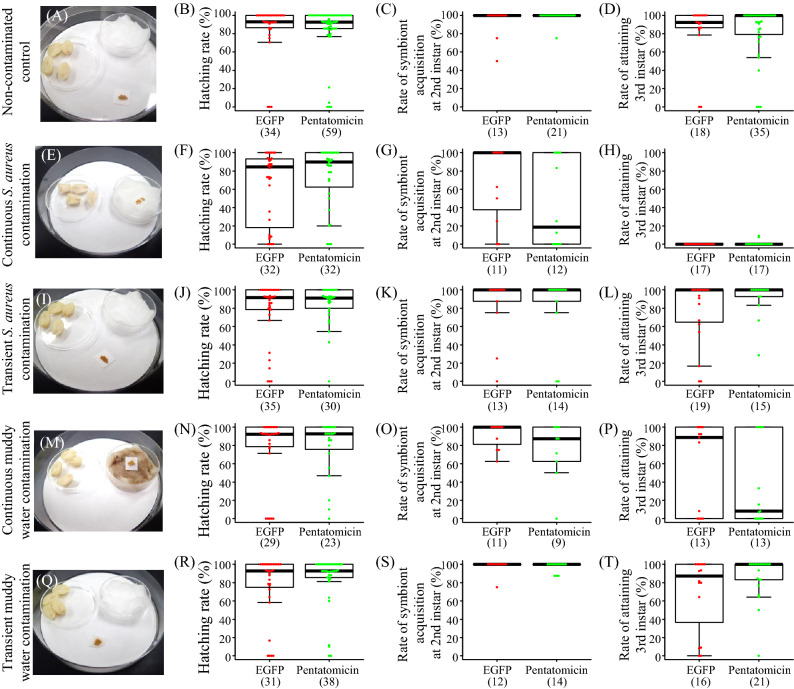
Effects of maternal RNAi of *Pentatomicin* on egg hatching, symbiont transmission and nymphal development under a variety of contaminating conditions. (**A**–**D**) Non-contaminated control, in which eggs were placed in a clean dish with sterilized water and peanuts. (**E**–**H**) Continuous *S. aureus* contamination, in which eggs were placed on liquid culture medium of *S. aureus* in a clean dish with sterilized peanuts. (**I**–**L**) Transient *S. aureus* contamination, in which eggs were soaked in liquid culture medium of *S. aureus* and then placed in a clean dish with sterilized water and peanuts. (**M**–**P**) Continuous muddy water contamination, in which eggs were placed on muddy water in a clean dish with sterilized peanuts. (**Q**–**T**) Transient muddy water contamination, in which eggs were soaked in muddy water and then placed in a clean dish with sterilized water and peanuts. (**A**,**E**,**I**,**M**,**Q**) Photographs of treated eggs. (**B**,**F**,**J**,**N**,**R**) Hatching rate. (**C**,**G**,**K**,**O**,**S**) Rate of symbiont acquisition at 2nd instar. (**D**,**H**,**L**,**P**,**T**) Rate of attaining 3rd instar. These rates were calculated in each egg mass and the number of egg masses is given in parentheses. In all the treatments, no statistically significant differences were observed between Pentatomicin-suppressed eggs and dsEGFP-injected control eggs for the hatching rates, symbiont acquisition rates, and rates of attaining 3rd instar (Mann–Whitney *U* test).

The high expression of *Pentatomicin* in eggs suggested the possibility that it may be involved in another biological role in *P. stali*. Note that, in *Drosophila*, some AMPs were reported to affect tumor inhibition, memory formation, and aging^45^. To examine the functional role of *Pentatomicin* in eggs, we performed maternal RNAi experiments as reported previously^30^. When dsRNA of *Pentatomicin* was injected into mated females, significantly lower expression was manifested in eggs laid by females that passed 3 days or more after injection, and the suppressed expression continued for 2 weeks thereafter (Fig. S7). Hatching rates of *Pentatomicin-*suppressed eggs were not significantly different from those of control eggs (Mann–Whitney *U* test; *P* = 0.68; Fig. 6A,B). The expression levels of *Pentatomicin* in the newborn nymphs were significantly lowered by the maternal RNAi treatment (Fig. S8). These results indicate that *Pentatomicin* is not essential for embryonic development of *P. stali*, although the possibility that a very low level of *Pentatomicin* is sufficient for ensuring embryonic development cannot be excluded.

### Biological role of Pentatomicin for ensuring vertical symbiont transmission?

Finally, we examined the possibility that *Pentatomicin* may contribute to successful vertical symbiont transmission in *P. stali*. It should be noted that the obligatory gut bacterial symbiont of *P. stali*, *Pantoea* sp. A, is Gram-negative and thus expected to be unaffected by *Pentatomicin* (Fig. 2). Upon oviposition, adult females of *P. stali* smear the symbiont-containing secretion onto the egg surface^24,25,28,29^, where the symbiotic bacteria persist for several days (ca. 5 days at 25 °C) until orally acquired by newborn nymphs. We hypothesized that *Pentatomicin* may protect the symbiotic bacteria on eggs from Gram-positive bacterial contaminants, which would otherwise outcompete the symbiont and lower the survival of the host insect. Symbiont-free nymphs of *P. stali* are known to suffer high mortality, especially in the 2nd instar, and severe developmental retardation^24,25,28^.

When the eggs were placed in a clean dish, suppression of *Pentatomicin* did not affect the hatching rate, the rate of successful symbiont acquisition at 2nd instar, and the rate of attaining 3rd instar within 10 days after egg hatching (Fig. 6A–D). Then, *Pentatomicin*-suppressed eggs were subjected to four different contaminating treatments: (i) placing the eggs continuously on liquid culture medium of *S. aureus* (Fig. 6E–H); (ii) soaking the eggs in liquid culture medium of *S. aureus* and then transferring them to a clean container (Fig. 6I–L); (iii) placing the eggs continuously on muddy water (Fig. 6M–P); (iv) soaking the eggs in muddy water and then placing them in a clean dish (Fig. 6Q–T). Among dsEGFP-injected controls, the continuous *S. aureus* treatment significantly reduced the hatching rate (Steel–Dwass test, *P* < 0.05; Fig. S9) and rates of reaching the 3^rd^-instar (Steel–Dwass test, *P* < 0.05; Fig. S9) in comparison with non-contaminated control, but the other treatments did not. In all the treatments, no statistically significant differences were observed between *Pentatomicin*-suppressed eggs and dsEGFP-injected control eggs for the hatching rates, symbiont acquisition rates, and rates of attaining 3^rd^ instar, although the medians did vary in some cases (Fig. 6). Taken together, the biological role of Pentatomicin in the early life stages was unclear. It is conceivable, though speculative, that *Pentatomicin* may confer resistance against some natural enemies like parasitic wasps, but further research is required to verify such hypotheses.

## Conclusion and perspective

We identified a new antimicrobial peptide, Pentatomicin, from the stinkbug *P. stali*. Pentatomicin-allied genes were found from hemipteran and coleopteran insects, a horseshoe crab, cyanobacteria, and proteobacteria. Using recombinant protein and conducting RNAi knockdown and quantitative RT-PCR, we uncovered the following properties of Pentatomicin. First, the antimicrobial activity of Pentatomicin was shown against an array of Gram-positive bacteria. Second, the expression of *Pentatomicin* increased not only upon septic shocks but also upon mock injection and saline injection, which is under the regulation of the Toll pathway, at least partly. Third, *Pentatomicin*-suppressed adult insects become more vulnerable to infection and pathology of Gram-positive *S. aureus*. Finally, high *Pentatomicin* expression was identified in eggs, which is atypical of conventional AMPs known from *Drosophila* and other insects, although the biological role of Pentatomicin in eggs is currently elusive. These findings extend our understanding of the diversity of AMPs, highlight a dynamic evolutionary aspect of AMPs, and provide a new insight into the innate immune components of insects. Conventionally, studies on insect immunity have been conducted mainly on holometabolous insects such as *Drosophila*^9,11,15,46,47^, *Manduca*^48,49^ and *Bombyx*^50,51^. For better understanding of the diversity of innate immunity, more studies on hemimetabolous insects are needed. In addition to *P. stali*, the bean bug *Riptortus pedestris*^52–56^ and the blood sucking bug *Rhodnius prolixus*^57–62^ would provide tractable models for understanding the innate immunity of hemimetabolous insects.

## Supplementary Information

Supplementary Information.

Supplementary Tables.

Supplementary Figures.

## Data Availability

Data sets generated or analyzed during this study are included in Supplementary Information files.
