# Supplementary material for: A new antimicrobial peptide, Pentatomicin, from the stinkbug Plautia stali
Source: Sci Rep. 2022 Oct 3;12:16503. doi: 10.1038/s41598-022-20427-w (PMC9529961; doi:10.1038/s41598-022-20427-w)
Supplement: Supplementary file 3 — Supplementary Figures. [file 41598_2022_20427_MOESM3_ESM.pdf]

A new antimicrobial peptide, Pentatomicin, from the stinkbug *Plautia stali*

Yudai Nishide, Keisuke Nagamine, Daisuke Kageyama, Minoru Moriyama, Ryo Futahashi, Takema Fukatsu

Supplementary figures

Fig. S1

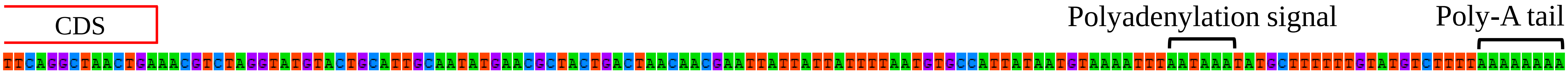

Supplementary Figure S1.
The nucleotide sequence of the *Pentatomicin* cDNA. On the downstream of the coding sequence, polyadenylation signal (AATAAA) and poly-A tail typical of eukaryotic genes are present.

Fig. S2

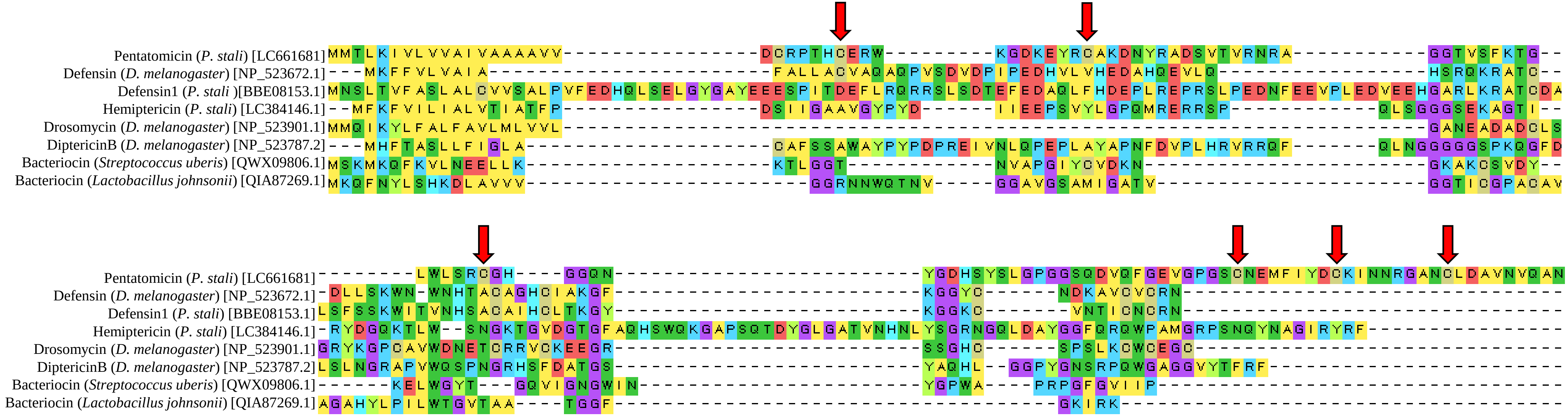

Supplementary Figure S2.
Comparison of amino acid sequences of Pentatomicin and other AMPs. Cysteine residues conserved among Pentatomicin and allies are indicated by red arrows.

Fig. S3

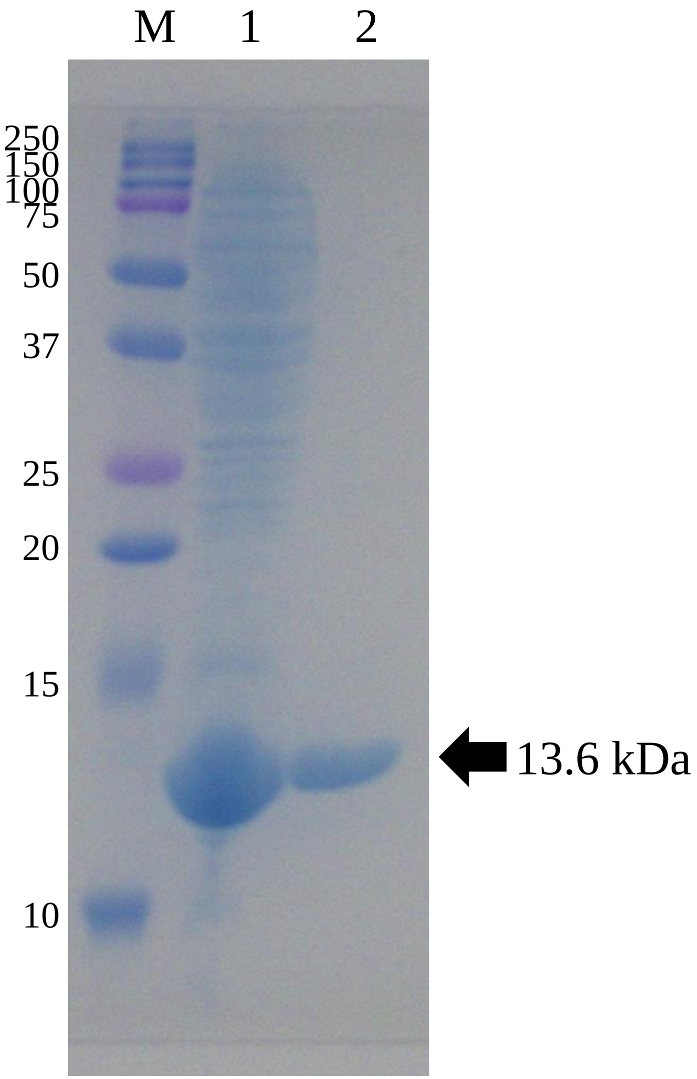

Supplementary Figure S3.
Expression and purification of recombinant Pentatomicin protein. Lane M, molecular size marker (Precision plus protein standards, BioRad, Hercules, USA); Lane 1, supernatant of recombinant BL21(DE3) bacterial lysate; Lane 2, Purified recombinant Pentatomicin protein. The deduced molecular mass of Pentatomicin (13.6 kDa) is indicated by an arrow.

Fig. S4

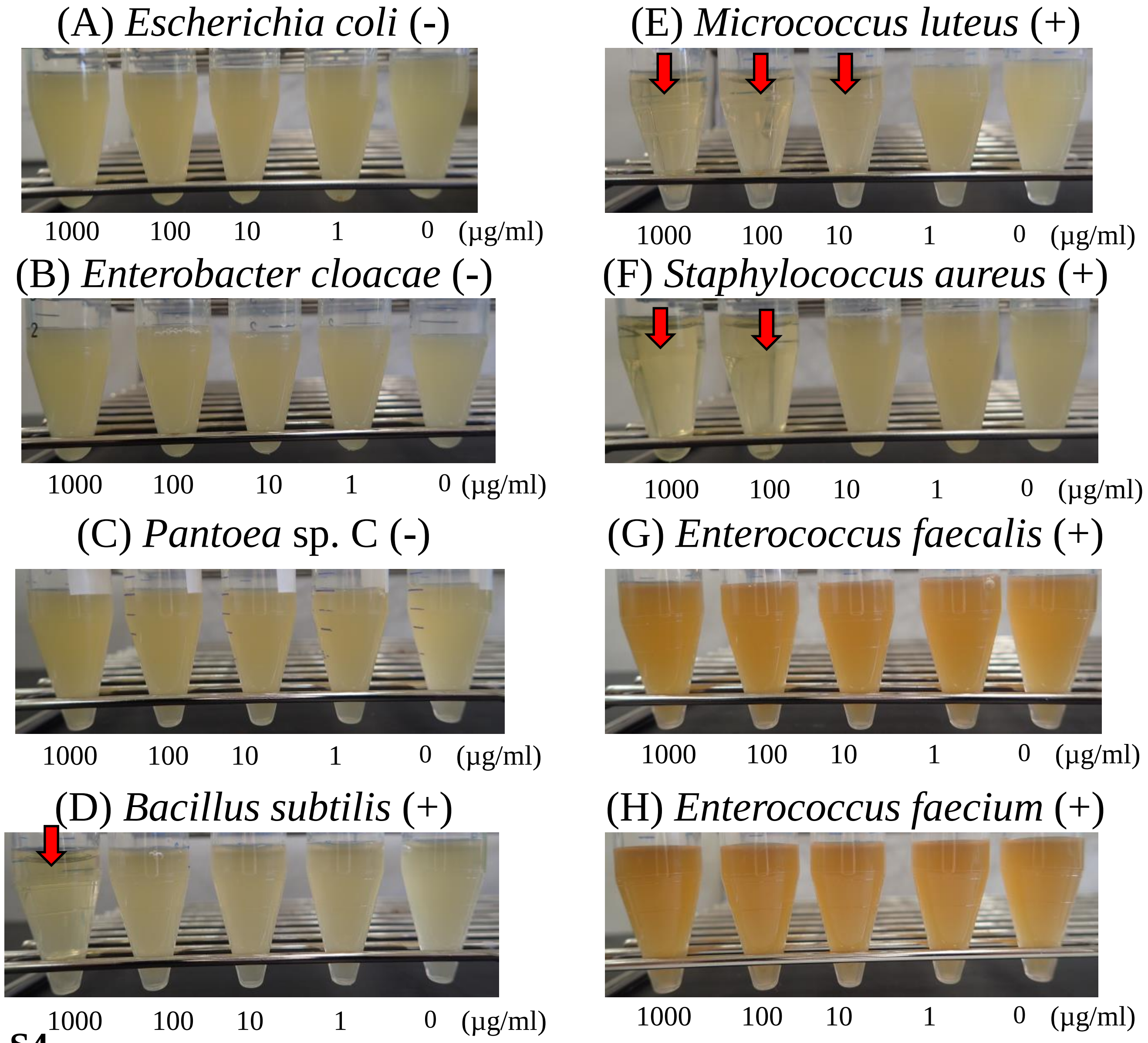

Supplementary Figure S4.

Antibacterial assay of recombinant Pentatomicin protein. (A) *Escherichia coli*. (B) *Enterobacter cloacae*. (C) *Pantoea* sp. C, the gut symbiont of *P. stali*. (D) *Bacillus subtilis*. (E) *Micrococcus luteus*. (F) *Staphylococcus aureus*. (G) *Enterococcus faecalis*. (H) *Enterococcus faecium*. Each bacterial liquid culture was supplemented with recombinant Pentatomicin and incubated at 25° C for 24 h. Pentatomicin doses in the tubes are shown below each panel. Red arrows indicate the tubes in which bacterial growth is suppressed.

Fig. S5

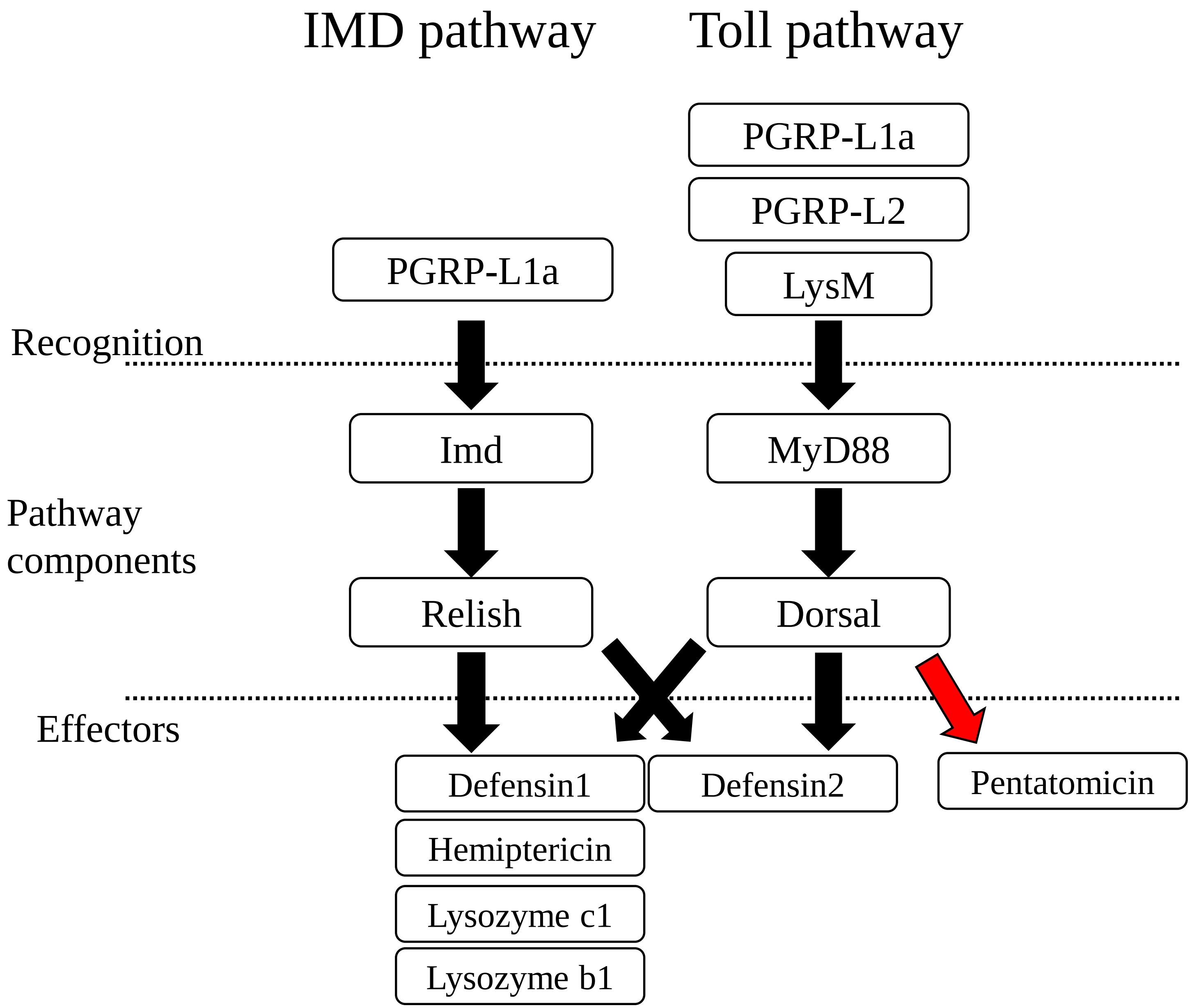

Supplementary Figure S5.
An updated schematic overview of IMD and Toll pathways in *P. stali*, which is based on Nishide *et al.*[32].

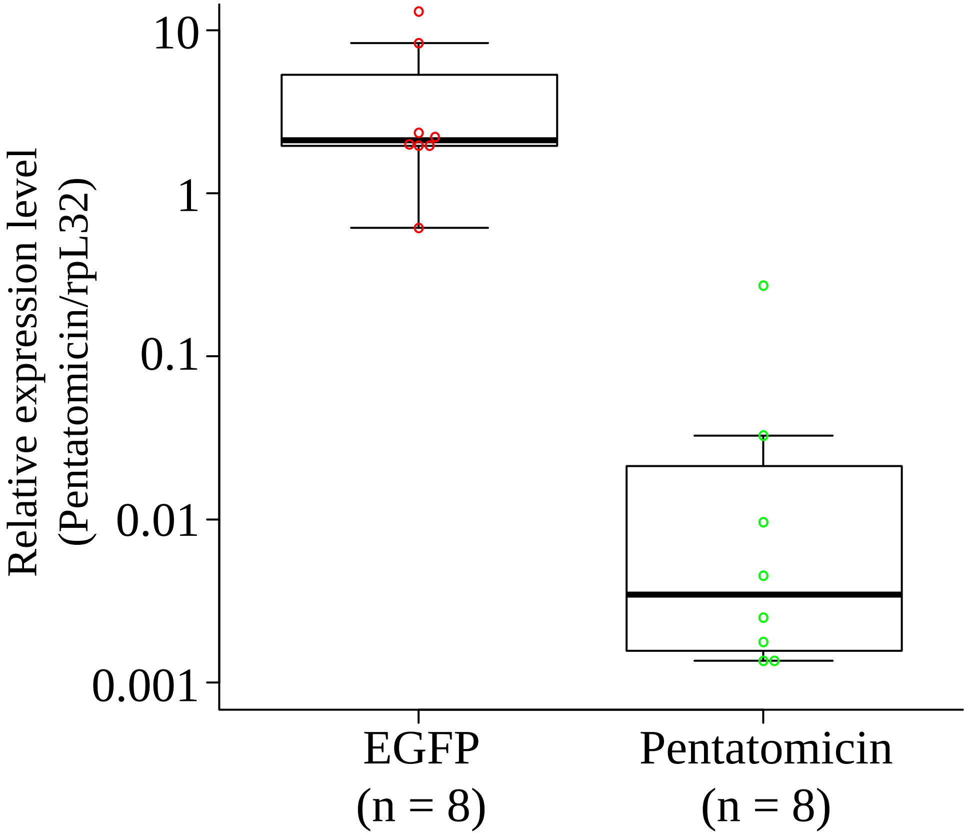

Supplementary Figure S6.

Effects of RNAi on the expression levels of *Pentatomicin* in adults. Adult females were injected with 10 ng of *E. coli* peptidoglycan three days after dsRNA injection and then subjected to RNA extraction on the following day. In comparison with dsEGFP injection, the difference is statistically significant (Mann Whitney U test; $P < 0.001$).

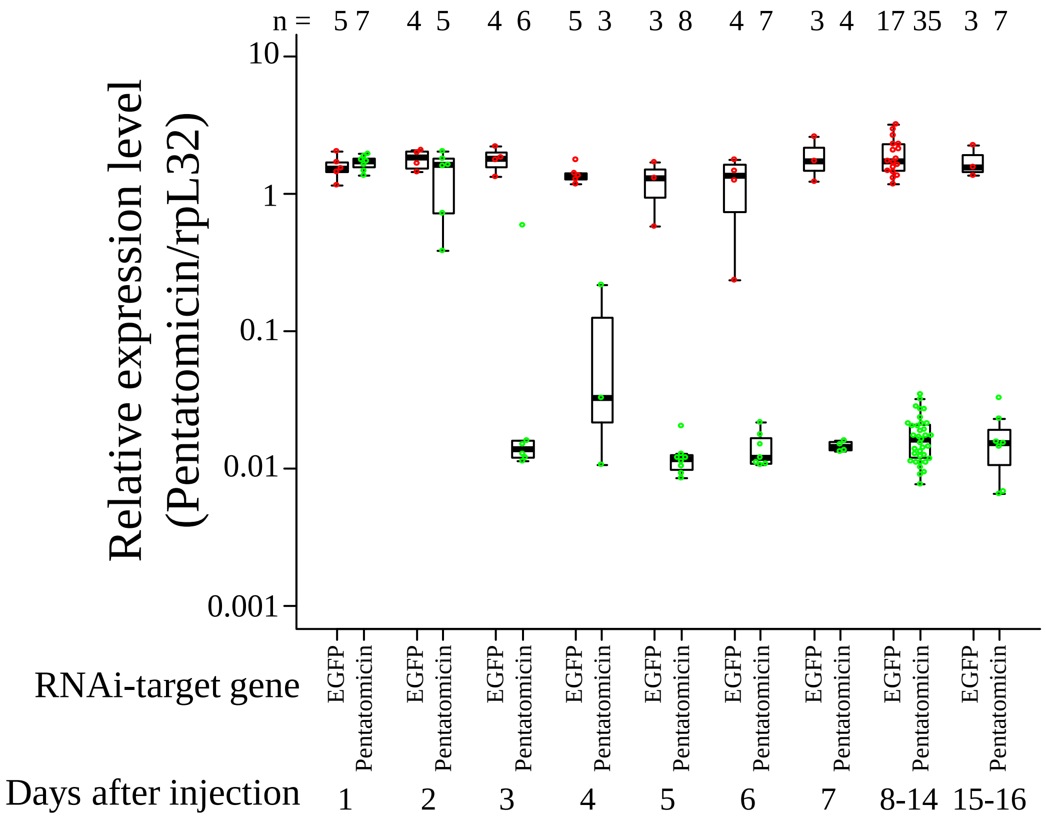

Supplementary Figure S7.

Effects of maternal RNAi on expression of *Pentatomicin* in eggs. Sexually mature females were injected with dsRNA of EGFP or *Pentatomicin*, their eggs were collected, and the expression levels of *Pentatomicin* in the eggs were measured by quantitative RT-PCR three days after oviposition.

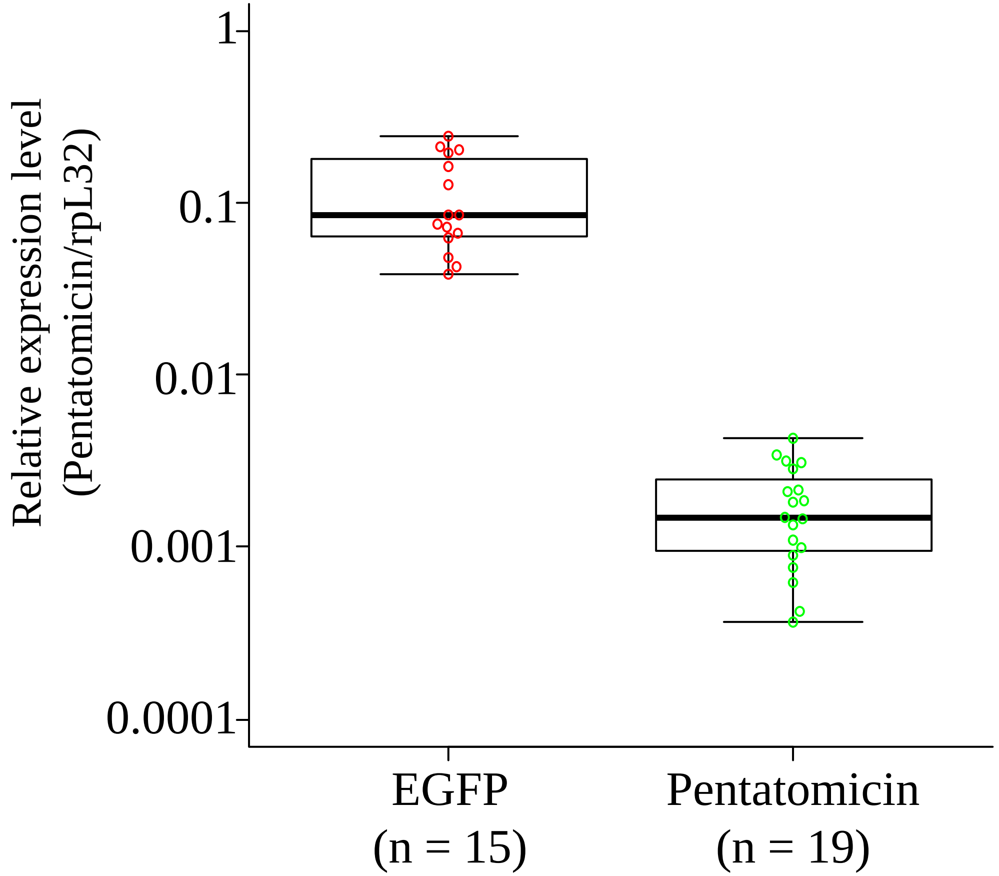

Supplementary Figure S8.

Effects of RNAi on the expression levels of *Pentatomicin* in 1st instar nymphs. The expression levels of *Pentatomicin* were measured for newly hatched nymphs from eggs laid by females more than five days after injection of dsRNA of EGFP or dsRNA of *Pentatomicin*. The difference is statistically significant (Mann Whitney U test; $P < 0.0001$).

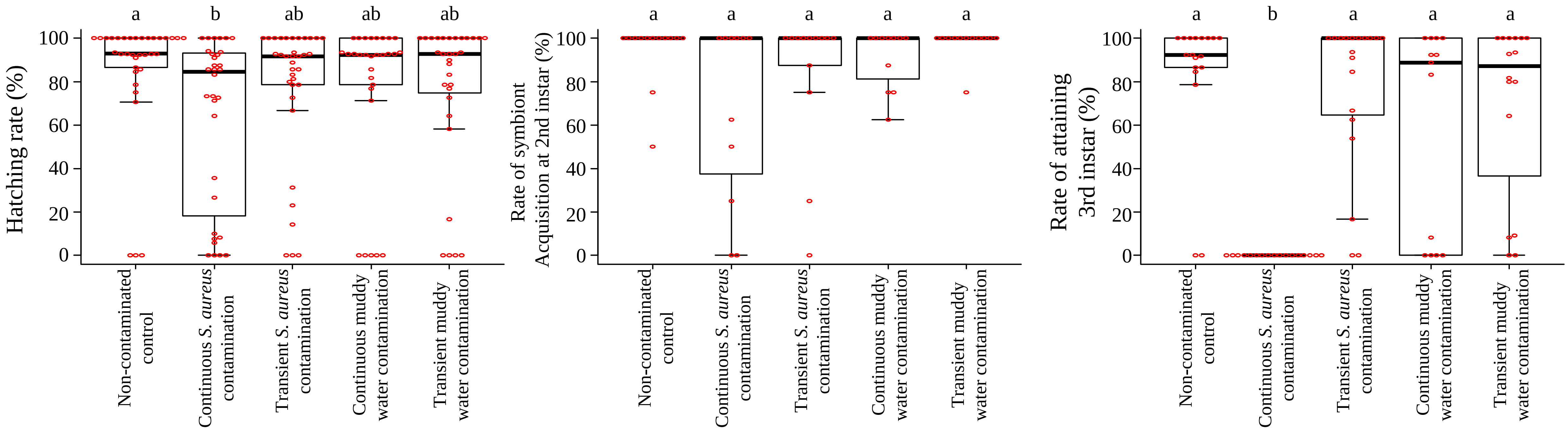

Supplementary Figure S9.

Comparison between EGFP-injected controls. Data are the same as EGFP-injected controls in Fig.6. Different letters indicate significant differences in Steel-Dwass test ($P < 0.05$).
